# A rare case of crescentic glomerulonephritis with monoclonal IgG deposits

**DOI:** 10.1080/0886022X.2021.1994997

**Published:** 2021-10-27

**Authors:** Xiaoyan Ma, Xun Zhou, Yi Wang, Min Tao, Lili Sheng, Shougang Zhuang, Na Liu

**Affiliations:** a Department of Nephrology, Shanghai East Hospital, Tongji University School of Medicine, Shanghai, China; b Department of Medicine, Rhode Island Hospital and Alpert Medical School, Brown University, Providence, RI, USA

Dear Editor,

A 62-year-old woman was admitted to our hospital because of edema of both lower limbs for more than 3 months and oliguria for 1 week. The patient’s laboratory test results were listed in Table 1. Her laboratory test results showed hemoglobin of 84 g/L, serum albumin of 25 g/L, blood urea nitrogen of 9.3 mmol/L, serum creatinine (Scr) of 687 µmol/L. The patient had oliguria (about 0.1 L/24H). Urinalysis showed proteinuria (protein excretion, 1.64 g/24 h). Serum C3 and C4 were within the normal range. Serum immunoglobulin (Ig) G level was reduced at 5.3 g/L. Protein electrophoresis showed monoclonal spikes (IgG κ type) in both her serum and urine. The serum-free κ chain was 229.25 mg/L and the free λ chain was 196.00 mg/L, both were higher than normal. The serum-free κ/λ chain ratio was 1.17. A serum cryoglobulin test was negative. Hepatitis B surface antigen, anti-HIV-1/2, and automated non-treponemal reagin test were all negative. Antinuclear antibodies, antineutrophil cytoplasmic antibodies, and anti-phospholipase A2 receptor (PLA2R) antibodies were also negative. The patient had no diagnosed history of metabolic disease, coronary heart disease, or liver disease.

**Table 1.** The patient’s laboratory test results on admission.

Variables	Value	Normal range
Hemoglobin (g/L)	84	115–150
Serum albumin (g/L)	25	40–55
Blood urea nitrogen (mmol/L)	9.3	3.1–8.8
Serum creatinine (μmol/L)	687	41–81
Serum cholesterol (mmol/L)	6.23	0.0–5.2
Urinary erythrocyte (/μL)	104.72	0–5
Serum C3 (g/L)	0.88	0.7–1.4
Serum C4 (g/L)	0.322	0.1–0.4
Serum IgG (g/L)	5.3	8.6–17.4
Rheumatoid factor (IU/ml)	1450	0–15.9
Serum free κ chain (mg/L)	229.25	3.3–19.1
Serum free λ chain (mg/L)	196	5.71–26.3
Serum-free κ/λ chain ratio	1.1696	0.26–1.65

An abdominal ultrasound examination showed normal renal size in both kidneys. We performed a renal biopsy for this patient after admission. Light microscopy examination showed membranoproliferative glomerulonephritis (MPGN) pattern and full of crescents (Figure 1(A–D)), and the percentage of renal interstitial fibrosis and tubule atrophy were about 8% under a light microscope. A renal biopsy specimen contained glomeruli (*n* = 37) and about 30 glomerulis were formed with crescents, mainly large cellular crescents. With the immunofluorescence examination, IgG and Kappa were deposited along the capillary wall and mesangial area (Figure 1(E,G)). There were no tubular deposits. Staining for IgG subtype was positive for IgG3 only, and negative for IgG1, IgG2, and IgG4 (Figure 1(F)). Electron microscopy examination revealed electron-dense deposits in the mesangial and subendothelial areas (Figure 2(A,B)). Bone marrow biopsy showed no obvious abnormality (Figure 3(A,B)). No plasma cells were observed in bone marrow smear, and the percentage of plasma cells observed in flow cytometry immunofluorescence analysis of bone marrow biopsy was 0.2%. Based on these findings, we completed the diagnosis of proliferative glomerulonephritis with monoclonal IgG deposits (PGNMID, IgG κ type).

**Figure 1.**
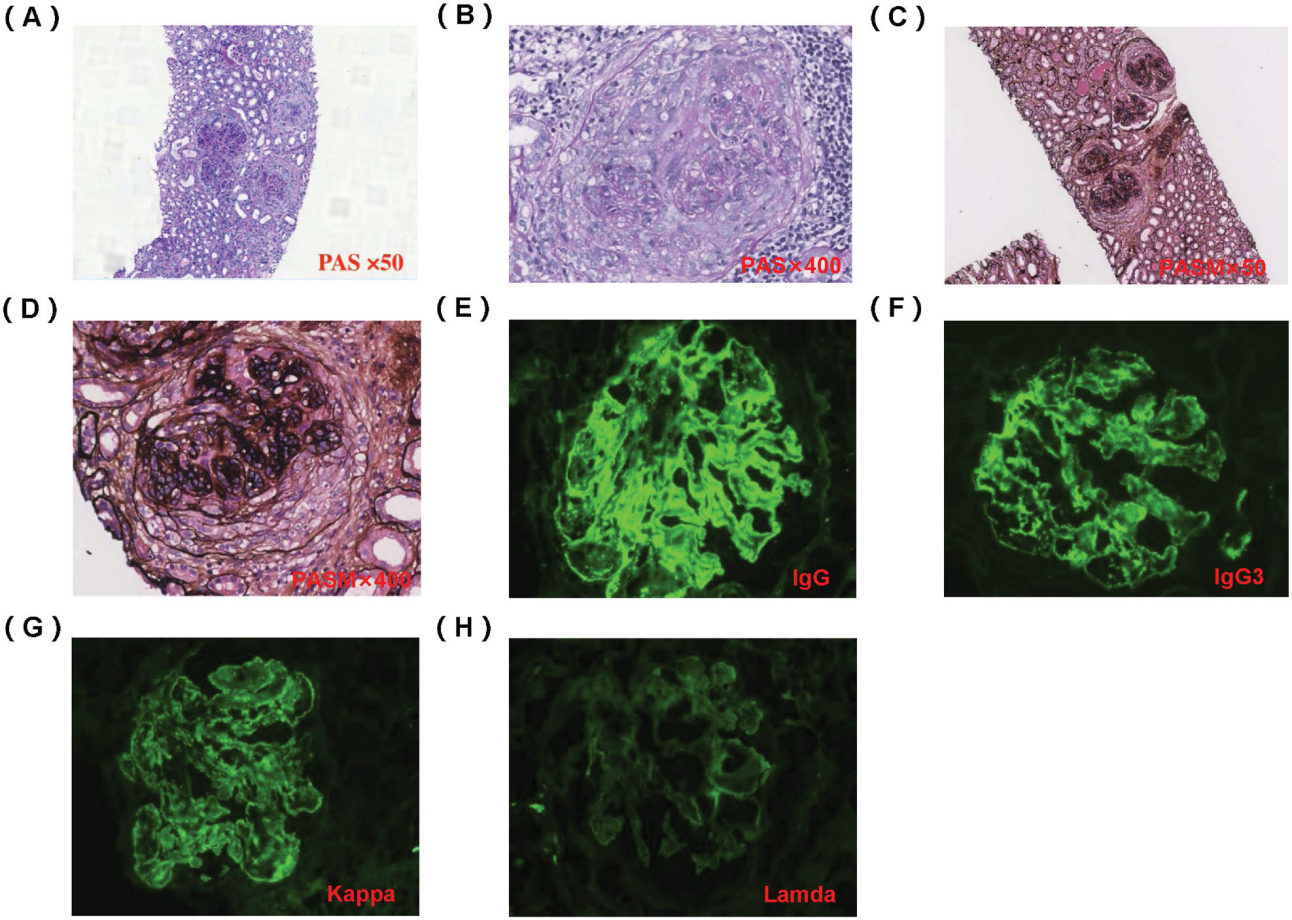
Pathology of renal biopsy. (A) Light microscopy (LM) shows cellular crescent, PAS staining × 50. (B) Large cellular crescent formation, PAS staining × 400. (C) LM showed an MPGN pattern and full of crescents, PASM staining × 50. (D) Cellular crescent formation and compressed capillary loops, PASM staining × 400. (E) Immunofluorescence shows granular IgG deposition along the capillary wall and in the mesangial area (×200). (F) Immunofluorescence shows IgG subtype is IgG3. (G) Immunofluorescence shows granular κ light chain deposition along the capillary wall and in the mesangial area (×200). (H) Immunofluorescence shows negativity for lambda light chain (×200).

**Figure 2.**
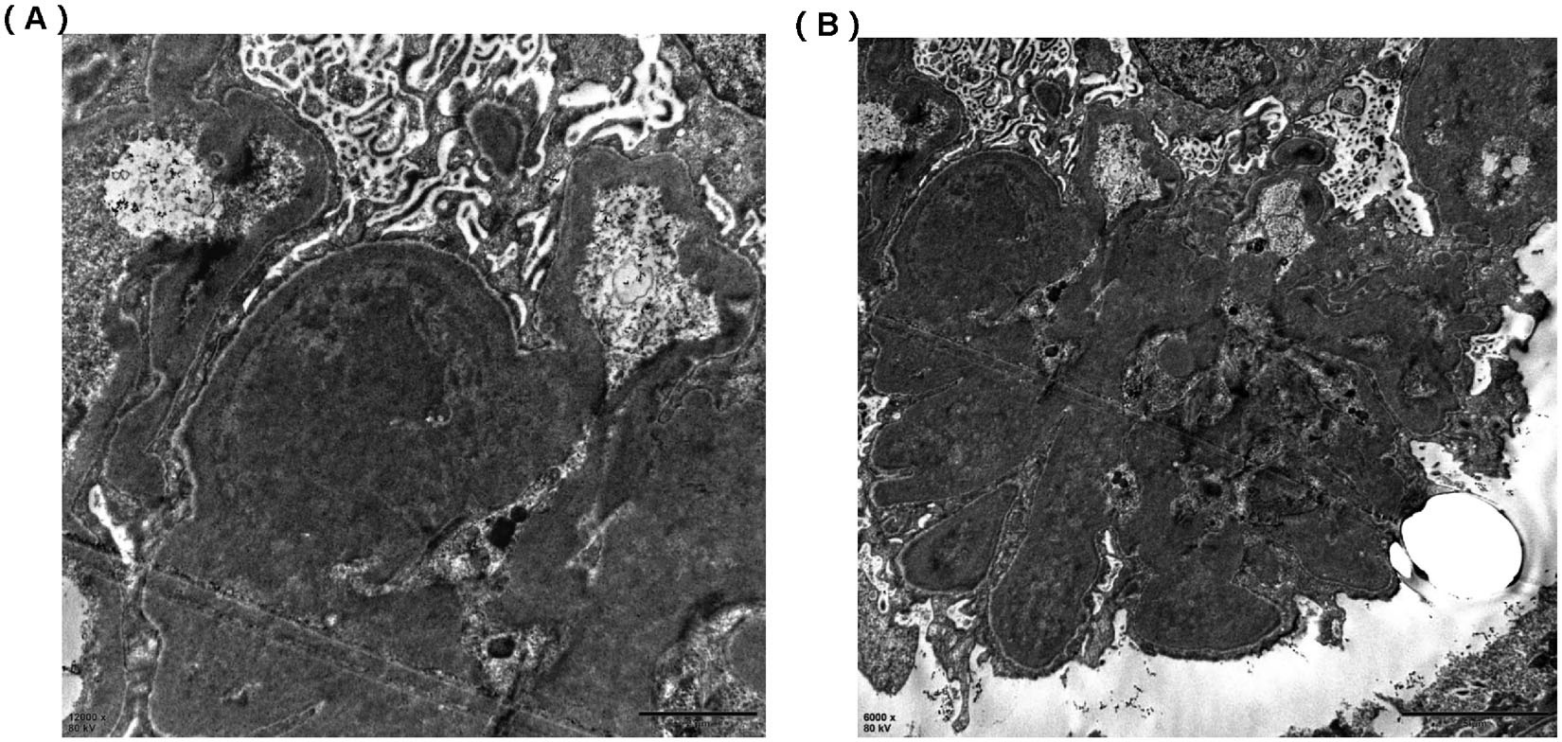
Electron microscope of renal biopsy specimens. (A) Electron microscope shows the electric-dense deposits in the mesangial area (×12,000). (B) Electron microscope shows the electric-dense deposits in subendothelial areas of glomeruli (×6000).

**Figure 3.**
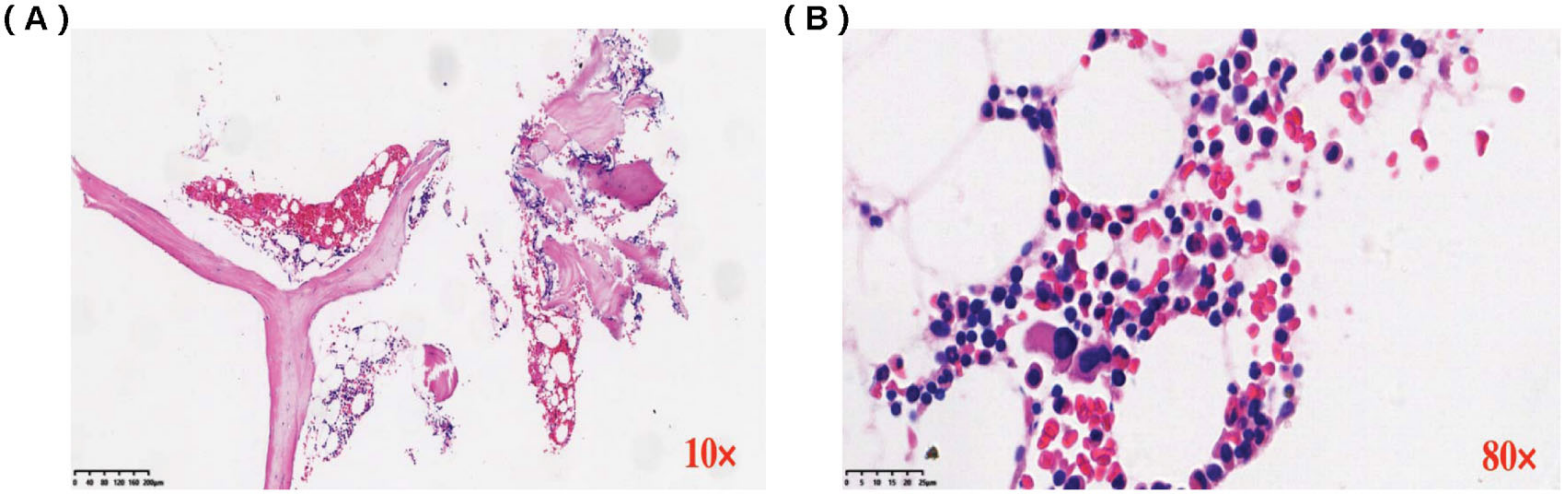
Bone marrow biopsy analysis. (A) Lymphocytes and plasma cells are scattered (×10). (B) There was no increase or aggregation of primitive/naive cells, lymphocytes, and plasma cells (×80).

Temporary hemodialysis was administered immediately after admission. After the renal biopsy, the patient received methylprednisolone therapy for 3 days (200 mg/d ivgtt) and gradually reduced the dose to 40 mg prednisone oral administration per day. In terms of immunosuppressants, we used cyclophosphamide with a cumulative dose of 2.0 g within 2 months. After the treatment of cyclophosphamide, the patient's renal function recovered, but proteinuria did not improve further. To prevent further disease progression, we used rituximab (500 mg) for multi-target and maintenance therapy. However, the monoclonal antibody therapy had to be terminated because the patient could not afford it. The specific time point is shown in Figure 4. The patient’s urine volume gradually recovered after 1 week of treatment, and she was able to stop dialysis after 2 weeks. One month later, the urine volume of the patient recovered to about 1 L/24 h, and Scr decreased from 687 µmol/L to 265 µmol/L. After 3 months of treatment, her renal function returned to normal. The patient’s last follow-up time was 18 August 2021, with serum albumin of 42.3 g/L, 24-h protein excretion of 0.34 g, and Scr of 118 μmol/L. We are still closely following up on this patient.

**Figure 4.**
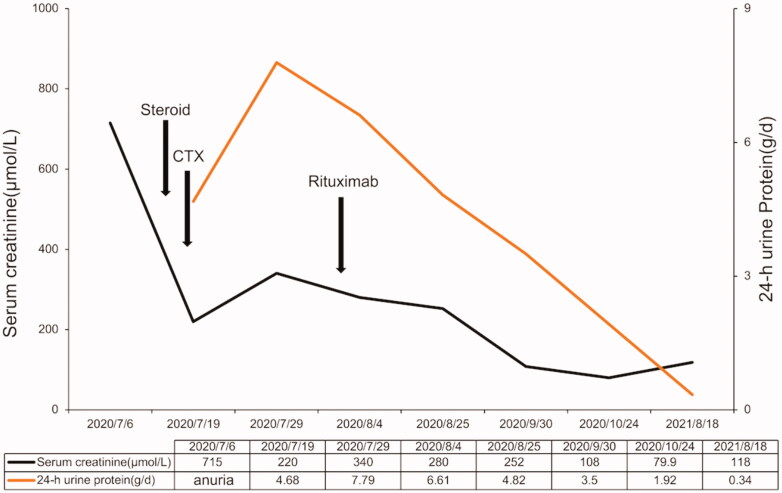
The patient’s treatment follow-up chart. The chart includes the time and dose of the patient’s steroid use and the timing of the use of immunosuppressants and the alterations of clinic parameters including urine protein and serum creatinine during the treatment.

## Discussion

PGNMID is a newly recognized disease characterized by glomerular monoclonal IgG deposition, which is first reported by Nasr et al. [1] in 2004, and its histopathological manifestations of renal biopsy show glomerular proliferative lesions. PGNMID is a rare kind of monoclonal gammopathy of renal significance with intact monoclonal IgG (single light-chain isotype and single γ heavy chain subtype) deposition [1]. The incidence of PGNMID diagnosis by autologous kidney biopsy is approximately 0.07%–0.17% [2,3]. MPGN (57%) and endocapillary proliferative glomerulonephritis (35%) were the most common light microscopy pathologic patterns [2]. The clinical manifestations of PGNMID are diverse. Almost all patients showed various degrees of proteinuria, hematuria and renal insufficiency. Due to its complexity, the pathogenesis of PGNMID is not fully understood and is easy to be misdiagnosed. The prognosis of PGNMID was poor, with 21.9% of patients progressing to end-stage renal disease (ESRD) and 37.5% patients with persistent renal insufficiency at a mean follow-up of 30.3 months [4]. In 2009, Nasr [2] reported several cases of PGNMID, about a quarter of these patients eventually progressed to ESRD. The common characteristics of progression to ESRD are associated with higher creatinine at biopsy, a higher percentage of global glomerulosclerosis and crescents [2]. In this series [2], crescents were present in 32.4% of cases, but most of them only affected a mean of 20% of glomeruli, and only 5.4% of these cases were presented with crescentic glomerulonephritis (crescent body proportion over 50%). The renal pathology of the patient in our case showed about 30 glomerulis (81.8%) were accompanied with crescent bodies mainly large cell crescents, which was rarely reported and predicting the disease was highly active.

Up to now, there are no effective methods to inhibit the deposition of Monoclonal immunoglobulin (MIg) in tissues or directly remove the deposition of MIg. Treatment of PGNMID is controversial due to limited cases and uncertain pathogenesis. It has been reported that partial remission can be achieved with immunosuppressants such as prednisolone, cyclophosphamide, mycophenolate mofetil and rituximab [2]. For M-spike negative patients, conservative treatment, including RAS inhibitors, is recommended for patients without the nephrotic syndrome. Steroids and cyclophosphamide are recommended in patients with nephrotic syndrome, patients who have failed treatment with RAS inhibitors, patients with reduced GFR, or patients whose biopsy features are suggestive of progression (e.g., crescents) [5]. Rituximab alone may have a better remission rate and better tolerance than steroids and cyclophosphamide [6]. Buxeda [7] reported that using anti-CD20 monoclonal antibodies to treat recurrent PGNMIDs with kidney allografts is effective. Nine relapsed patients only received anti-CD20 antibody (rituximab) treatment, and both their glomerular filtration rate and proteinuria were improved. The patient in our case was a 62-year-old Chinese woman who presented with acute renal failure and nephrotic syndrome, and about 30 glomerulis (81.8%) were accompanied with crescent bodies mainly large cell crescents. Japanese scholars reported a very similar case [8] that the pathology of the patient showed that almost all glomerulis showing cellular and circumferential global crescents. They treated the patient with methylprednisolone 500 mg for 3 days and subsequent oral administration of 40 mg/day of prednisolone, but no improvement of renal function was observed after 2 weeks. Intravenous cyclophosphamide and 6 days of plasmapheresis treatment were additionally performed, but severe proteinuria persisted and his serum creatinine level remained a high level, and the patient had to continue to maintain hemodialysis [8]. In our case, We chose steroids and cyclophosphamide to induce remission since her crescentic glomerulonephritis (Figure 4). Rituximab was also used in combination with multi-target therapy and maintenance therapy during treatment. A three-month short-term observation suggested that the patient had achieved partial remission. The patient’s last follow-up time was 18 August 2021, with serum albumin of 42.3 g/L, 24-h protein excretion of 0.34 g, and Scr of 118 μmol/L. The patient is still followed closely in the clinic, including M-spike monitoring.

## Conclusion

We report a PGNMID patient presented with acute renal failure and nephrotic syndrome, and her renal biopsy suggested crescentric glomerulonephritis. The patient achieved partial remission rapidly after the treatment of steroid and cyclophosphamide, suggesting that early administration of immunosuppressive therapy may contribute to reverse progression.
